# ALDH1A1 regulates postsynaptic μ–opioid receptor expression in dorsal striatal projection neurons and mitigates dyskinesia through transsynaptic retinoic acid signaling

**DOI:** 10.1038/s41598-019-40326-x

**Published:** 2019-03-05

**Authors:** Jing Pan, Jia Yu, Lixin Sun, Chengsong Xie, Lisa Chang, Junbing Wu, Sarah Hawes, Sara Saez–Atienzar, Wang Zheng, Justin Kung, Jinhui Ding, Weidong Le, Shengdi Chen, Huaibin Cai

**Affiliations:** 1 Department of Neurology, Ruijin Hospital Affiliated to Shanghai Jiao Tong University School of Medicine, Shanghai, 200025 P. R. China; 2 Transgenic Section, Laboratory of Neurogenetics, National Institute on Aging, National Institutes of Health, Bethesda, MD 20892 USA; 3 Institute for Geriatrics and Rehabilitation, Beijing Geriatric Hospital, Beijing University of Chinese Medicine, Beijing, 100095 P. R. China; 4 Bioinformatics Core, Laboratory of Neurogenetics, National Institute on Aging, National Institutes of Health, Bethesda, MD 20892 USA; 5 Clinical Research Center on Neurological Diseases, the First Affiliated Hospital, Dalian Medical University, Dalian, 116011 P. R. China; 6 Present Address: Children’s National Medical Center, Washington, D.C. USA; 7 Present Address: University of Maryland, School of Medicine, Baltimore, Maryland USA

## Abstract

Aldehyde dehydrogenase 1A1 (ALDH1A1), a retinoic acid (RA) synthase, is selectively expressed by the nigrostriatal dopaminergic (nDA) neurons that preferentially degenerate in Parkinson’s disease (PD). ALDH1A1–positive axons mainly project to the dorsal striatum. However, whether ALDH1A1 and its products regulate the activity of postsynaptic striatal neurons is unclear. Here we show that μ–type opioid receptor (MOR1) levels were severely decreased in the dorsal striatum of postnatal and adult *Aldh1a1* knockout mice, whereas dietary supplement of RA restores its expression. Furthermore, RA treatment also upregulates striatal MOR1 levels and signaling and alleviates L-DOPA–induced dyskinetic movements in *pituitary homeobox 3* (*Pitx3*)–deficient mice that lack of ALDH1A1–expressing nDA neurons. Therefore, our findings demonstrate that ALDH1A1–synthesized RA is required for postsynaptic MOR1 expression in the postnatal and adult dorsal striatum, supporting potential therapeutic benefits of RA supplementation in moderating L-DOPA–induced dyskinesia.

## Introduction

Postmortem brains from patients with Parkinson’s disease (PD) display regionally selective loss of nigrostriatal dopaminergic (nDA) neurons, preferentially in the ventral tier of *substantia nigra pars compacta* (SNpc)^1–3^. Aldehyde dehydrogenase 1A1 (ALDH1A1), a multifunctional enzyme, is predominantly expressed by this PD-vulnerable, ventral tier nDA neuron subpopulation^2,4^. ALDH1A1 mediates the synthesis of retinoic acid (RA)^5^, which activates retinoic acid receptors (RARα, RARβ, RARγ) and promotes transcription of various downstream genes critical for neuronal development, patterning, and survival^6^. In DA neurons, ALDH1A1 also converts the cytotoxic dopamine intermediate 3, 4-dihydroxyphenylacetaldehyde (DOPAL) into the less reactive acid forms, and thereby protects the DA neurons^7,8^. Additionally, ALDH1A1 synthesizes inhibitory neurotransmitter γ-aminobutyric acid (GABA) in DA neurons, where co-release of dopamine and GABA regulates alcohol consumption and preference^9^. ALDH1A1 proteins are enriched in both soma and axon terminals of nDA neurons, which mainly innervate the rostral dorsal striatum^10^. ALDH1A1 expression is substantially reduced in the remaining nDA neurons in PD brains^11–13^. However, little is known about the effect of ALDH1A1 downregulation on the organization and function of postsynaptic striatal neurons in PD.

Dorsal striatum can be divided into two complementary compartments named striosome (or “patch”) and matrix, based on differential gene expression, developmental origin, and connectivity^14,15^. The μ–type opioid receptor **(**MOR1) is mainly expressed by the striatal projection neurons (SPNs) in the striosomes^16–18^. MOR1 belongs to a subfamily of G protein–coupled receptors that activate the downstream G_i_ or G_o_–mediated inhibitory signaling pathways and modulate reward seeking behaviors^18,19^. Alterations of MOR1 expression and functions are associated with PD and alcoholism^20,21^. However, how MOR1 is regulated in the dorsal striatum and whether the altered MOR1 levels observed in PD contribute to pathogenesis are unclear.

Current mainstream anti–PD drugs like dopamine precursor L-3,4-dihydroxypheylalanine (L-DOPA) are effective in improving patients’ movements; however, prolonged administration of L-DOPA can cause L-DOPA–induced dyskinesia (LID) as well as impulsive control disorders^22,23^. Increasing studies are focusing on the causes and treatments of LID. However, new mechanistic insights and therapeutic agents are still needed to improve patients’ treatment and life quality.

To investigate the relationship between ALDH1A1 and postsynaptic MOR1 expression, we examined MOR1 levels in *Aldh1a1*^−/−^ mice. We observed a substantial reduction of MOR1 protein and mRNA levels in the dorsal striatum of postnatal and adult *Aldh1a1*^−/−^ mice. RA content in the dorsal striatum of *Aldh1a1*^−/−^ mice was also reduced. We then treated *Aldh1a1*^−/−^ mice for seven days with RA supplemented diet, which restored MOR1 levels. ALDH1A1 is under transcriptional control of pituitary homeobox 3 (PITX3) in the midbrain DA neurons^24^. *Pitx3* spontaneous knockout (*Pitx3*^ak/ak^) mice show PD–like severe loss of ALDH1A1–positive nDA neurons^24–26^. *Pitx3*^ak/ak^ mice also exhibit L-DOPA–induced abnormal dyskinetic climbing movements^27,28^. We thereby examined the regulatory role of RA in MOR1 signaling in the nDA neuron–depleted *Pitx3*^ak/ak^ mice and found RA treatment upregulated MOR1 levels, mitigated L-DOPA–induced dyskinetic movements, and reduced a hyperactivation of PKA/ERK signaling seen in the dorsal striatum of *Pitx3*^ak/ak^ mice, similar to *Aldh1a1* knockouts. Together, these results reveal a previously unknown function of ALDH1A1 in upregulating postsynaptic μ-opioid receptor levels in the dorsal striosomes through transsynaptic RA signaling, thereby mitigating L-DOPA–induced dyskinetic movements.

## Results

### *Aldh1a1*–deficiency causes severe reduction of MOR1 expression in the postsynaptic striatal neurons

**Figure 1:**
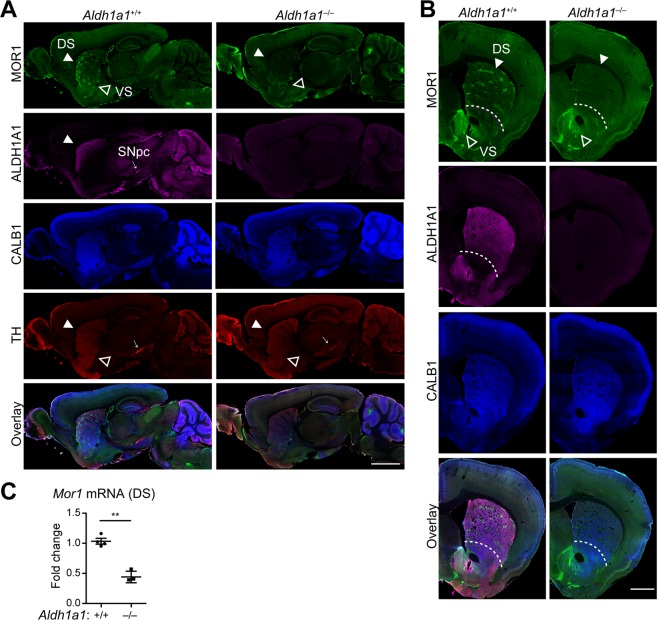
Severe reduction of MOR1 expression in the postsynaptic striatal neurons of postnatal and adult *Aldh1a1*^−/−^ mice. (**A**) Representative images show MOR1 (green), ALDH1A1 (purple), CALB1 (blue) and TH (red) co-staining in the sagittal sections of 3–month–old *Aldh1a1*^−/−^ and littermate *Aldh1a1*^+/+^ mice (n > 5 per genotype). Solid arrowheads indicate the MOR1–positive striosomes in the dorsal striatum (DS). Open arrowheads point to the ventral striatum (VS) and adjacent structures. Scale bar: 1500 µm. (**B**) Representative images show MOR1 (green), ALDH1A1 (purple), CALB1 (blue) and TH (red) co-staining in the coronal sections of 3–month–old *Aldh1a1*^−/−^ and *Aldh1a1*^+/+^ mice (n > 5 per genotype). Arrows indicate the MOR1–positive striosomes in the dDS. Arrowheads point to VS. Dashed lines outline the segregation between DS and VS for image analyses. Scale bar: 1000 µm. (**C**) Scatter plot depicts the expression of *Mor1* mRNAs in the DS of 3–month–old *Aldh1a1*^+/+^ (n = 4) and *Aldh1a1*^−/−^ (n = 3) mice by qRT-PCR. Data were presented as mean ± SEM. Unpaired t test, t = 6.016, df = 5. **P < 0.01.

ALDH1A1–positive axon fibers from the ventral midbrain nDA neurons project to the rostral dorsal portion of dorsal striatum (Fig. 1A, Additional File 1: Fig. S1). To examine the impact of *Aldh1a1*–deficiency on MOR1 expression, we co-stained series of brain sections from 3–month–old *Aldh1a1*^−/−^ mice and wild type (*Aldh1a1*^+/+^) controls with antibodies against MOR1 and matrix marker calbindin (CALB1). Substantial reduction of MOR1 staining was observed in the rostral dorsal striatum of *Aldh1a1*^−/−^ mice, whereas no apparent alteration of CALB1 expression was found (Fig. 1A). The decrease of MOR1 levels was most profound in regions of dorsal striatum that normally receive heavy innervation from ALDH1A1–positive DA axon fibers in wild type mice (Fig. 1B; Additional File 1: Figs S1, S2 for series of images across the striatum). Normally the ventral striatum is also intensively innervated by ALDH1A1–positive fibers from DA neurons located in the ventral tegmental area (VTA); however, only a modest reduction of MOR1 expression was observed in ventral striatum of *Aldh1a1*^−/−^ mice (Fig. 1B, Additional File 1: Fig. S2). The deficit in dorsal striatal MOR1 existed at the transcriptional level, as *Mor1* mRNA was found to be reduced in the dorsal striatum of 3–month–old *Aldh1a1*^−/−^ mice (Fig. 1C). By contrast, no obvious changes of mRNA expression were detected in other opioid receptors, endogenous opioid peptides, and retinoid receptor RARβ in the dorsal striatum of *Aldh1a1*^−/−^ mice (Fig. 1C, Additional File 1: Fig. S3A–C). Nor did *Aldh1a1*–deficiency affect *Mor1* mRNA expression in prefrontal cortex or hippocampus (Additional File 1: Fig. S3D,E). Collectively, these results reveal a previously undescribed function of ALDH1A1 in maintaining the expression of MOR1 in postsynaptic SPNs preferentially localized in the dorsal striatum. The same reduction of dorsal striatal MOR1 levels was seen in male and female *Aldh1a1*^−/−^ mice; therefore, we did not segregate by sex in the rest of the study.

### ALDH1A1 is required for postsynaptic MOR1 expression in the dorsal striatum of postnatal and adult mice

**Figure 2:**
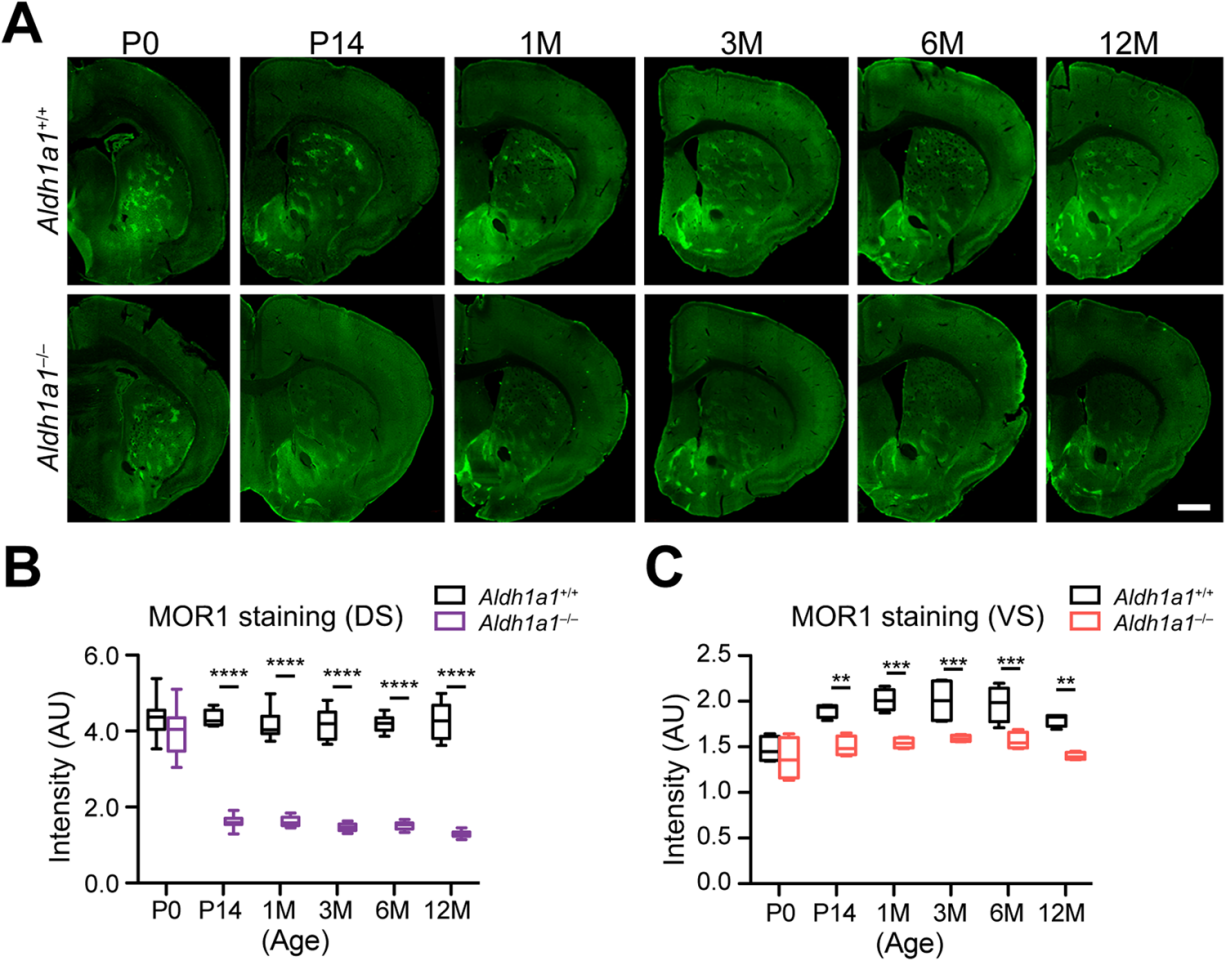
A loss of MOR1 expression in dorsal striatum occurs in postnatal and adult *Aldh1a1*^−/−^ mice. (**A**) Representative images show MOR1 staining in the selective striatal coronal sections of *Aldh1a1*^+/+^ and *Aldh1a1*^−/−^ mice at different age. Scale bar: 1000 μm. (**B,C**) Box and whiskers plots (min to max) show the signal intensities of MOR1 staining in the DS (**B**) and VS (**C**) of *Aldh1a1*^+/+^ and *Aldh1a1*^−/−^ mice at different ages (n = 5 animals per genotype per age group, 3 sequential coronal sections between Bregma 0.84–1.10 per animal). Data were presented as mean ± SEM. Two-way ANOVA was used for statistical analysis, followed by Bonferroni’s *post hoc* tests. Genotype factor: DS, F_(1, 111)_ = 1347.42, ****p < 0.0001; VS, F_(1, 24)_ = 61.20, ****p < 0.0001.

To investigate when the reduction of MOR1 expression occurs in the dorsal striatum, we examined striatal sections prepared from postnatal day 0 (P0) to 12–month–old mice. We found that, whereas postnatal MOR1 expression remained constant in *Aldh1a1*^+/+^ mice, a dramatic, nonlinear reduction of MOR1 expression appeared in the dorsal striatum of *Aldh1a1*^−/−^ mice, with a rapid decrease between postnatal day 0 (P0) and P14, followed by a gradual reduction continuing from P14 to 12 months of age (Fig. 2A,B). In the ventral striatum, MOR1 levels were markedly increased in the ventral striatum of *Aldh1a1*^+/+^ mice after P14, but they did not show a similar alteration in the *Aldh1a1*^−/−^ mice (Fig. 2A,C). These data demonstrate that ALDH1A1 facilitates MOR1 expression in the striatum, but it is only required to maintain postsynaptic MOR1 expression in the dorsal striatum of postnatal and adult mice.

### A loss of ALDH1A1 does not alter the compartmentation of dorsal striatum

**Figure 3:**
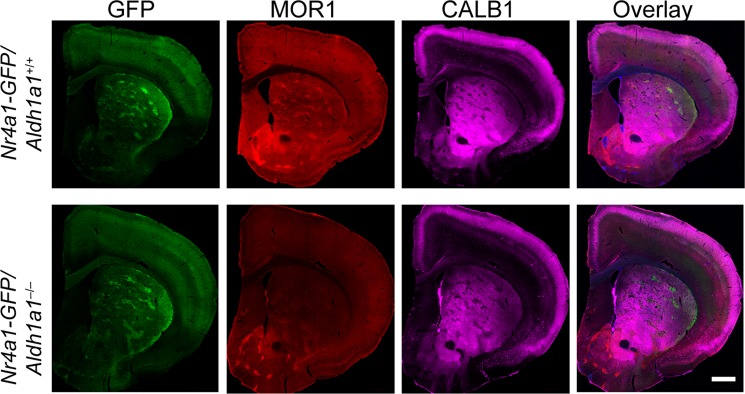
ALDH1A1–deficiency does not affect the organization of striosome and matrix compartments. Representative images show GFP (green), MOR1 (red), CALB1 (purple) staining in the 3-month-old Nr4a1-GFP transgenic mice with *Aldh1a1*^+/+^ and *Aldh1a1*^−/−^ genetic background (n = 6 per genotype). Scale bar: 1000 μm.

*Aldh1a1*^−/−^ mice showed normal expression and distribution of matrix marker CALB1 in the dorsal striatum (Fig. 1A,B), suggesting that a loss of ALDH1A1 does not affect the overall striatal compartmentation between striosome and matrix. To further test this hypothesis, we crossbred Nr4a1–GFP transgenic mice, which selectively express the green fluorescent protein (GFP) in the striosomes under the transcriptional control of nuclear receptor 4a1 (Nr4a1)^29^, in the *Aldh1a1*^+/+^ and *Aldh1a1*^−/−^ backgrounds and examined the GFP signals in the dorsal striatum. We observed comparable GFP signals marking striosomes within 3–month–old Nr4a1–GFP/*Aldh1a1*^+/+^ and Nr4a1–GFP/*Aldh1a1*^−/−^ mice, in contrast to a severe reduction of MOR1 staining in the *Aldh1a1*^−/−^ genetic background (Fig. 3). These results demonstrate that a lack of ALDH1A1 disrupts MOR1 expression in the striosomes but spares striosome and matrix compartmentation in the dorsal striatum.

### Acute knock-down of *Aldh1a1* in nDA neurons reduces MOR1 expression in the *Aldh1a1*^+/+^ SPNs

**Figure 4:**
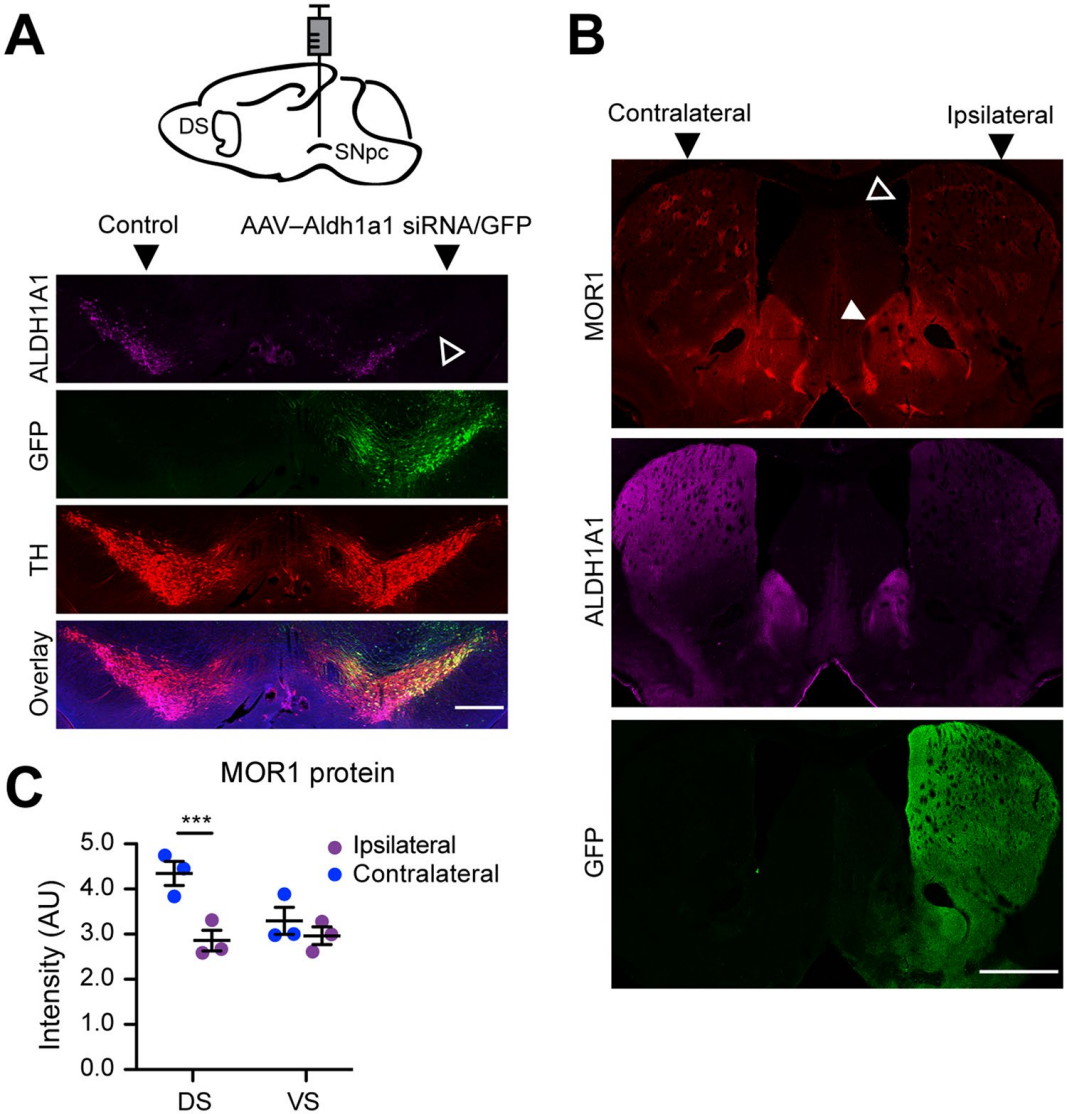
Acute inhibition of *Aldh1a1* in nDA neurons reduces MOR1 expression in the *Aldh1a1*^+/+^ SPNs. (**A**) Cartoon illustrates the needle placement for stereotaxic injection of AAVs in the SNpc. Representative images show GFP (green), ALDH1A1 (purple), and TH (red) staining at DLS of 3-month-old *Aldh1a1*^+/+^ mice (n = 3) injected with AAVs co–expressing *Aldh1a1* siRNA and GFP in one hemisphere. Open arrowhead points to the part of SNpc with severe reduction of ALDH1A1 staining. Scale bar: 500 µm. (**B**) Representative images show MOR1 (red) ALDH1A1 (purple), and GFP (green) staining at the striatum of the same animal in (**A**). Open arrowhead points to the DS with severe reduction of MOR staining. Solid arrowhead marks the rather normal MOR1 staining in the ventral striatum and adjacent regions. Scale bar: 1000 µm. (**C**) Scatter plot compares the signal intensities of MOR1 staining in the DS and VS of the ipsilateral and contralateral injection sites (n = 3 animals, 3 sequential coronal sections per animal). Data were presented as mean ± SEM. Two-way ANOVA followed by Sidak’s multiple comparisons test: DS, t_12_ = 5.035, ***p < 0.001; VS, t_12_ = 1.112, ns.

Although ALDH1A1 is predominantly expressed by the ventral midbrain DA neuron subpopulations in the brain, to exclude the possibility of ALDH1A1 from the other sources in regulating MOR1 expression in the dorsal striatum, we selectively suppressed the expression of ALDH1A1 in the nDA neurons by stereotaxic injection of recombinant adeno-associated viruses expressing both *Aldh1a1* siRNA and GFP (AAV–Aldh1a1 siRNA/GFP)^9^ into the SNpc of 3–month–old *Aldh1a1*^+/+^ mice. In correlation with the diminished expression of ALDH1A1 in the soma and axon terminals of AAV–infected nDA neurons, MOR1 expression was reduced in the dorsal striatum (Fig. 4A–C). In controls, injection of only GFP-expressing AAVs did not affect MOR1 expression (data not shown). Together, these data confirm that ALDH1A1 proteins in the dorsal striatum originate from the nDA axon terminals, and further establish the function of presynaptic ALDH1A1 in maintaining postsynaptic MOR1 levels in postnatal and adult SPNs.

### RA supplementation restores MOR1 expression in the dorsal striatum of *Aldh1a1*^−/−^ mice

**Figure 5:**
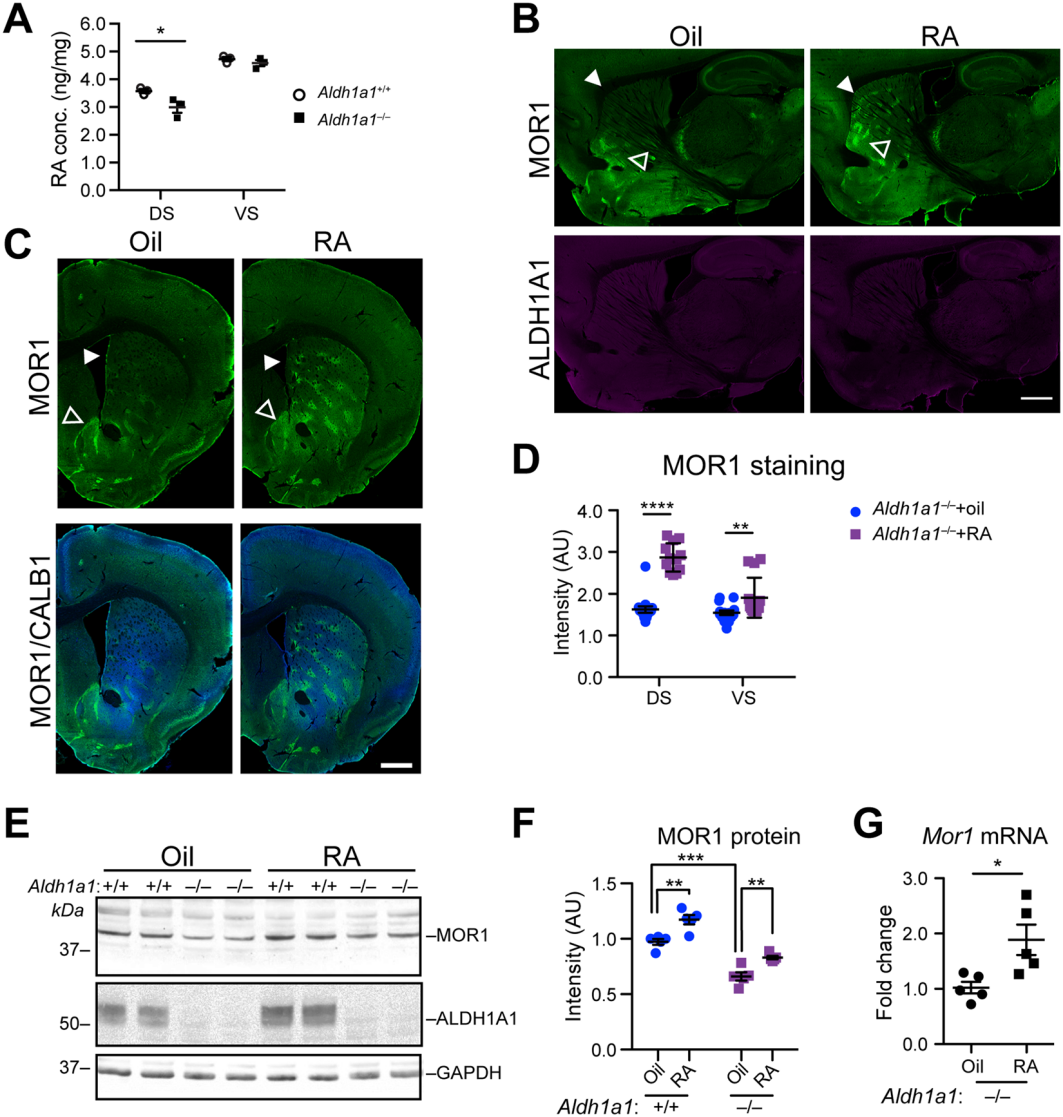
Dietary supplement of RA restores MOR1 expression in the dorsal striatum of *Aldh1a1*^−/−^ mice. (**A**) Scatter plot shows the endogenous RA concentration in the DS and VS of 12-month-old *Aldh1a1*^+/+^ and *Aldh1a1*^−/−^ mice (n = 3 animals per genotype). Data were presented as mean ± SEM; *p < 0.05. Unpaired t-test was used for statistical analysis. (**B**) Representative images show MOR1 (green) and ALDH1A1 (purple) co-staining in the sagittal sections of 3–month–old *Aldh1a1*^−/−^ mice (n = 4 per group) after treated with vehicle corn oil or RA for seven days. Solid arrowheads point to the dorsal striatum. Open arrowheads mark the ventral striatum and adjacent structures. Scale bar: 500 µm. (**C**) Representative images show MOR1 (green), and CALB1 (blue) co-staining in the coronal sections of 3–month–old *Aldh1a1*^−/−^ mice after treated with corn oil or RA for seven days. Solid arrowheads point to the dorsal striatum. Open arrowheads mark the BNST. Scale bar: 1000 µm. (**D**) Scatter plot shows the signal intensities of MOR1 staining in the DS and VS of 3–month–old *Aldh1a1*^−/−^ mice after treated with corn oil or RA for seven days (n = 4 animals per genotype per age group, 3 sequential coronal sections between Bregma 0.84–1.10 per animal). Data were presented as mean ± SEM. Two-way ANOVA followed by Sidak’s multiple comparisons test: DS, t = 10.38, ****p < 0.0001; VS, t = 3.150, **p < 0.01. (**E**) Western blots show MOR1 and ALDH1A1 expression in the dorsal striatum of 3–month–old *Aldh1a1*^+/+^ and *Aldh1a1*^−/−^ mice treated with oil or RA for seven days. GAPDH was used as the loading control. (**F**) Scatter plot depicts the changes of MOR1 protein expression in the dorsal striatal tissues (n = 5 animals per genotype per treatment). Data were presented as mean ± SEM. One-way ANOVA followed by Tukey’s multiple comparison test: q_+/+oil vs −/−oil_ = 9.774, ***p < 0.001; q^+/+^_oil vs_^+/+^_RA_ = 6.314, **p < 0.01; q^+/+^_oil vs −/−RA_ = 4.471, *p < 0.05; q_−/−oil vs −/−RA_ = 5.303, **p < 0.01; q^+/+^_RA vs −/−RA_ = 10.78, ***p < 0.001. (**G**) Scatter plot shows *Mor1* mRNA levels in the DS of *Aldh1a1*^−/−^ mice after treated with corn oil or RA for seven days (n = 5 animals per treatment). Data were presented as mean ± SEM. Unpaired t test, t_8_ = 2.923, p = 0.0192.

Given that RA is a product of ALDH1A1 with a recognized role in striosomal neurogenesis^30^, and that we detected a significant reduction of RA concentration in dorsal striatum of 12-month-old *Aldh1a1*^−/−^ mice compared to age-matched controls (Fig. 5A), we tested the effect of RA replenishment in *Aldh1a1*^−/−^ mice. We treated *Aldh1a1*^+/+^ and *Aldh1a1*^−/−^ mice with diets supplemented with RA (dissolved in corn oil and mixed into diet at a final concentration of 0.25 mg/g food) or else the solvent corn oil alone. RA treatment for seven days increased MOR1 expression levels and restored the normal expression pattern of MOR1 in the dorsal striatum of *Aldh1a1*^−/−^ mice at 3, 6, and 12 months of age (Fig. 5B–D, Additional File 1: Fig. S4A). Corn oil alone had no effect on MOR1 expression. RA–mediated increase of MOR1 protein and mRNA expression was also observed by western blot and qRT-PCR (Fig. 5E–G). Notably, RA treatment also elevated the expression of MOR1 in the dorsal striatum of *Aldh1a1*^+/+^ mice (Fig. 5E,F; Additional File 1: Fig. S4B,C), supporting a more general function of RA in promoting MOR1 expression in the dorsal striatum. By contrast, RA did not alter the expression of other opioid receptor expression in the dorsal striatum (Additional File 1: Fig. S4D,E). These data demonstrate that RA produced by presynaptic ALDH1A1 is essential in maintaining the postsynaptic expression of MOR1 in the dorsal striatum of postnatal and adult mice.

### RA supplementation does not alter motor activity of *Aldh1a1*^−/−^ mice

**Figure 6:**
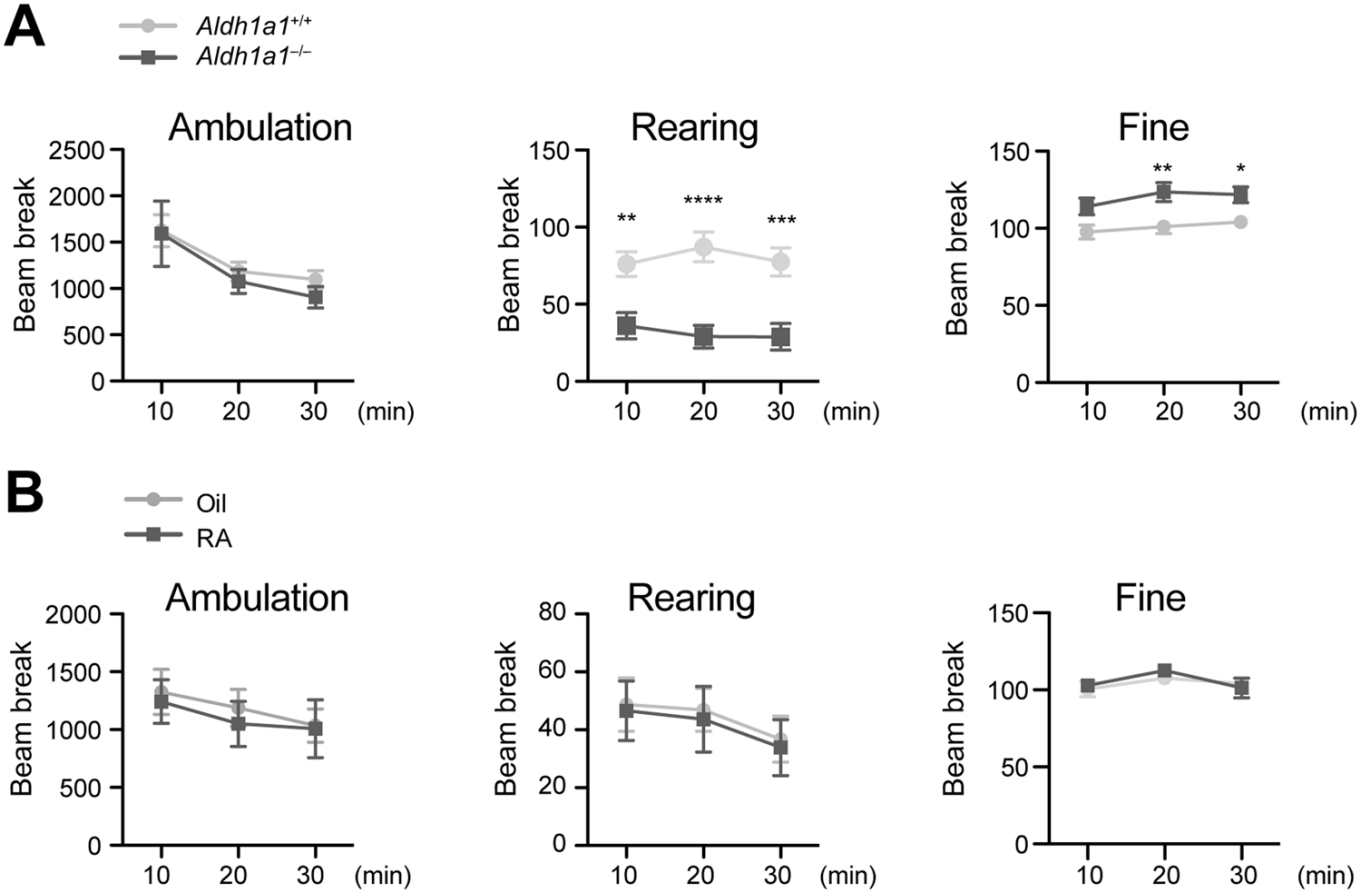
Open-field tests of *Aldh1a1*^+/+^ and *Aldh1a1*^−/−^ mice. (**A**) Line graphs quantify the ambulation, rearing, and fine movement of 3–month–old *Aldh1a1*^+/+^ (n = 14) and *Aldh1a1*^−/−^ (n = 14) mice. Data were presented as mean ± SEM. Two-way ANOVA followed by Bonferroni multiple comparisons test: ambulation, F_(1, 26)_ = 1.00, p = 0.3559; rearing, F_(1, 26)_ = 17.95, p = 0.0003; fine, F_(1, 26)_ = 10.51, p = 0.0032. (**B**) Open-field tests of 3–month–old *Aldh1a1*^−/−^ mice treated with oil (n = 10) or RA (n = 12). Data were presented as mean ± SEM. Two-way ANOVA followed by Bonferroni multiple comparisons test: ambulation, F_(1, 60)_ = 0.26, p = 0.9596; rearing, F_(1, 60)_ = 0.91, p = 0.7299; fine, F_(1, 60)_ = 0.15, p = 0.7022.

*Aldh1a1*^−/−^ mice exhibited normal walking in the Open–field test^9^, but showed decreased rearing and increased fine movement (Fig. 6A). Despite restoring MOR1 levels to dorsal striatum, RA treatment did not rescue the abnormal rearing and fine movements in *Aldh1a1*^−/−^ mice (Fig. 6B), suggesting that MOR1 signaling may not regulate general locomotion.

### RA supplementation increases MOR1 expression in the dorsal striatum of *Pitx3*^ak/ak^ mice

**Figure 7:**
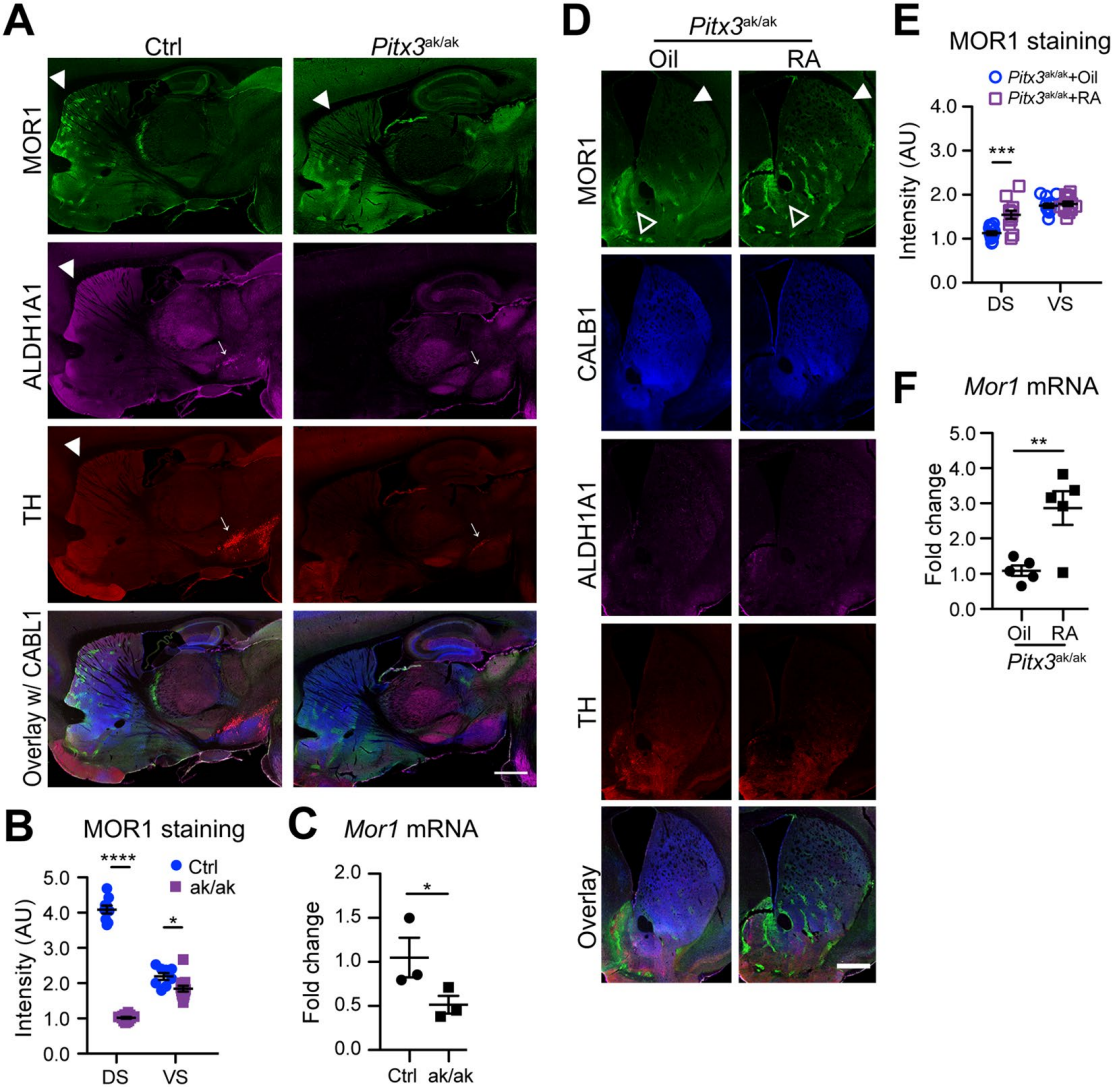
RA administration partially rescues MOR1 expression in the dorsal striatum of *Pitx3*^ak/ak^ mice. (**A**) Representative images show MOR1 (green), ALDH1A1 (purple), TH (red), and CALB1 (blue) co-staining in the sagittal sections of 3–month–old *Pitx3*^ak/ak^ and littermate control (Ctrl) mice. Solid arrowheads indicate the MOR1–positive striosomes in the dorsal striatum. Arrows point to the midbrain. Scale bar: 1500 µm. (**B**) Scatter plot shows the signal intensities of MOR1 staining in the DS and VS of 3–month–old control and *Pitx3*^ak/ak^ mice (n = 3 animals per genotype per age group, 3 sequential coronal sections per animal). Data were presented as mean ± SEM. Two-way ANOVA followed by Sidak’s multiple comparisons test: dDS, t_56_ = 26.40, ****p < 0.0001; VS, t_56_ = 2.952, *p < 0.05. (**C**) Scatter plot depicts the expression of *Mor1* mRNA in the DS of 3–month–old control (n = 3) and *Pitx3*^ak/ak^ (n = 3) mice by qRT-PCR. Data were presented as mean ± SEM. Unpaired t test, t_4_ = 2.168, p = 0.0480. (**D**) Representative images show MOR1 (green), CALB1 (blue), ALDH1A1 (purple), TH (red) co-staining in the coronal sections of 3–month–old *Pitx3*^ak/ak^ mice after treated with corn oil or RA for seven days. Solid arrowheads point to the DS. Open arrowheads mark the VS. Scale bar: 1000 µm. (**E**) Scatter plot shows the signal intensities of MOR1 staining in the DS and VS of 3–month–old *Pitx3*^ak/ak^ mice after treated with corn oil or RA for seven days (n = 3 animals per genotype per age group, 3 sequential coronal sections per animal). Data were presented as mean ± SEM. Two-way ANOVA followed by Sidak’s multiple comparisons test: DS, t_70_ = 4.275, ***p < 0.001; VS, t_70_ = 0.4344, ns. (**F**) Scatter plot shows *Mor1* mRNA levels in the DS of *Pitx3*^ak/ak^ mice after treated with corn oil or RA for seven days (n = 5 animals per treatment). Data were presented as mean ± SEM. Unpaired t test, t8 = 2.923, p = 0.0192.

The expression of *Aldh1a1* is under transcriptional control of PITX3 in the nDA neurons^24^. PITX3 is critical for the terminal differentiation and survival of nDA neurons, and *Pitx3*–deficiency causes developmental defects of nDA neurons and depletion of dopamine in the dorsal striatum^31^. Only a few ALDH1A1–positive neurons were present in the SNpc, while more surviving neurons were found in the VTA^24^. Concomitantly, ALDH1A1, TH and MOR1 staining were drastically reduced in the dorsal striatum of 3–month–old *Pitx3*^ak/ak^ mice, while CALB1 expression was not affected (Fig. 7A,B), indicating that a lack of PITX3 does not alter the compartmentation of dorsal striatum, but downregulates MOR1 expression in the striosomes similar to *Aldh1a1* knockout. Meanwhile, *Mor1* mRNA levels were also severely decreased in the dorsal striatum of *Pitx3*^ak/ak^ mice (Fig. 7C). The loss of MOR1 expression also occurred postnatally, since the level and pattern of MOR1 expression appeared normal in the dorsal striatum of P0 *Pitx3*^ak/ak^ pups (Additional File 1: Fig. S5). Consistent with the previous finding that no nDA neurons exist to project to the dorsal striatum of *Pitx3*^ak/ak^ mice^24,31^, stereotaxic injection of ALDH1A1–expressing AAVs in the SNpc failed to deliver ALDH1A1 proteins to the striatum and rescue MOR1 expression (data not shown). By contrast, ectopic expression of ALDH1A1 directly in the dorsal striatum enhanced the MOR1 expression in the ALDH1A1–expressing striatal neurons (Additional File 1: Fig. S6), further supporting a role of ALDH1A1 and its products in regulating MOR1 expression in SPNs. We then followed the same protocol to treat *Pitx3*^ak/ak^ mice with RA for seven days. RA administration markedly improved but did not completely restore the expression levels of MOR1 in the dorsal striatum of *Pitx3*^ak/ak^ mice (Fig. 7D,E). A similar increase of *Mor1* mRNA was also detected in the RA-treated animals (Fig. 7F). Together, these data demonstrate that RA remains effective in promoting MOR1 expression in the dorsal striatum of *Pitx3*^ak/ak^ mice even in the absence of nDA axon innervation.

A lack of *Pitx3* also led to a substantial decrease of *Dor1* and increase of *Kor1* mRNA expression in the dorsal striatum of 3–month–old *Pitx3*^ak/ak^ mice (Additional File 1: Fig. S7A,B). However, the application of RA did not normalize transcription levels of these two opioid receptors (Additional File 1: Fig. S7A,B). In addition to elevating MOR1, RA treatment reversed an elevation of MOR1 ligand *Penk* mRNA expression found in the dorsal striatum of 3–month–old *Pitx3*^ak/ak^ mice (Additional File 1: Fig. S7C). On the other hand, *Pitx3* deficiency and RA treatment had no effect on the expression of another opioid ligand, *Pdyn* mRNA levels (Additional File 1: Fig. S7D). These results reveal more dynamic alterations of endogenous opioid receptors and ligands expression in the dDS of *Pitx3*^ak/ak^ mice compared to the *Aldh1a1*^−/−^ animals (Additional File 1: Fig. S3). In addition, RA administration enhanced the expression of *RAR*β mRNAs in the dorsal striatum of *Pitx3*^ak/ak^ mice (Additional File 1: Fig. S7E).

### RA supplementation reduces L-DOPA–induced repetitive climbing movements in *Pitx3*^ak/ak^ mice

**Figure 8:**
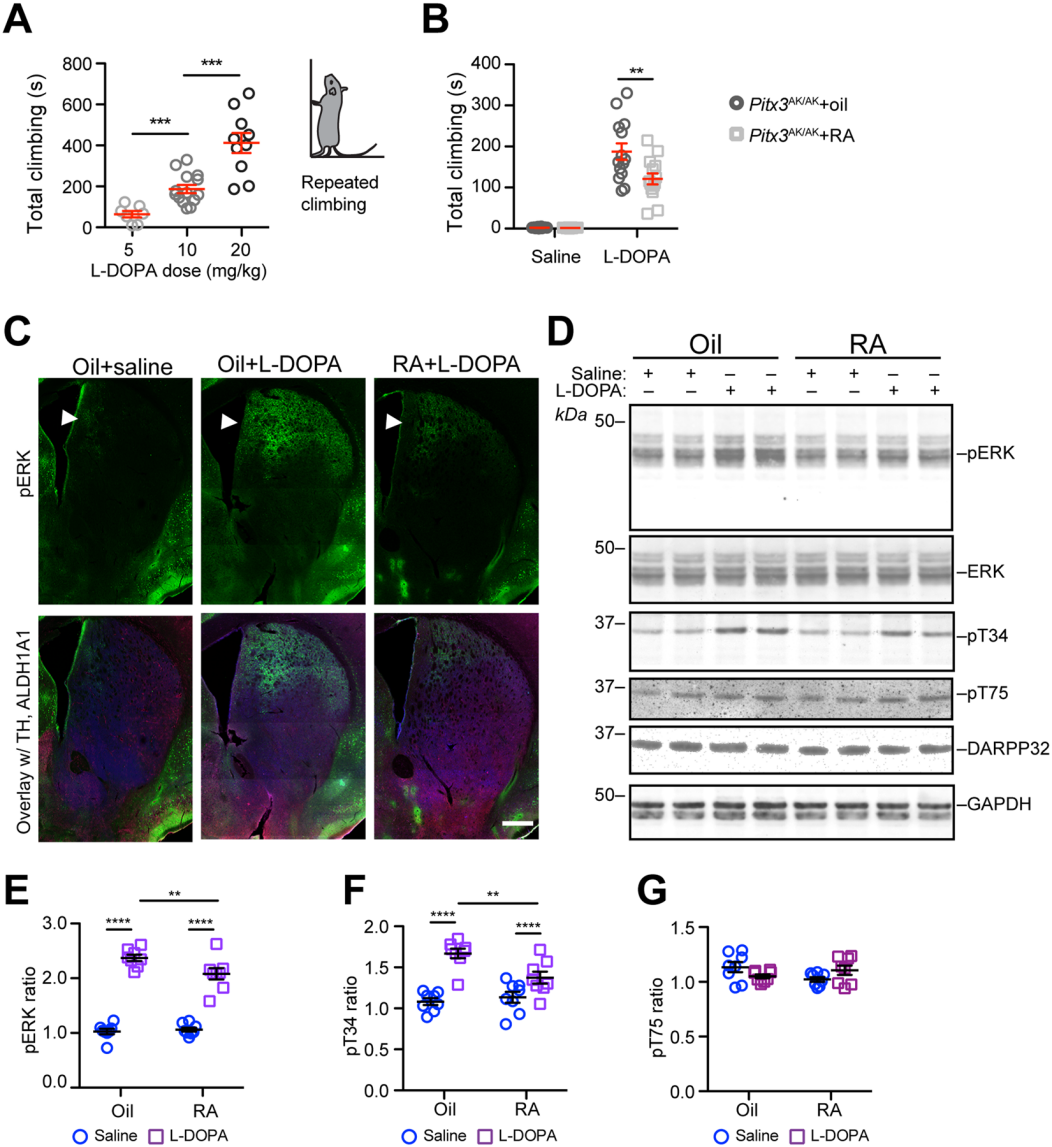
RA treatment reduces L-DOPA–induced repetitive climbing movements in *Pitx3*^ak/ak^ mice and suppresses ERK and PKA signaling in the dorsal striatum. (**A**) Scatter plot shows the dose effects of L-DOPA administration in inducing the stereotypic, repetitive climbing movements in male *Pitx3*^ak/ak^ mice at 3 to 4 months of age (n = 7–14 animals per dose). Data were presented as mean ± SEM. One-way ANOVA followed by Tukey’s multiple comparison test: q_5vs10_ = 3.662, **p < 0.05; q_5vs20_ = 9.686, ***p < 0.001; q_10vs20_ = 7.434, ***p < 0.001. ***P < 0.001. The right cartoon highlights the vertical posture adopted by L-DOPA–treated in *Pitx3*^ak/ak^ mice with 2 to 4 paws on the wall of container. **(B**) Male *Pitx3*^ak/ak^ mice at 3 to 4 months of age (n = 14 animals per group) was pretreated with RA or corn oil for seven days before the administration of L-DOPA at 10 mg/kg bodyweight or saline. Data were presented as mean ± SEM. Two-way ANOVA followed by Sidak’s multiple comparisons test: Oil/saline vs RA/saline, t_52_ = 0.0021, ns; Oil/saline vs Oil/L-DOPA, t_52_ = 10.97, ****p < 0.0001; RA/saline vs RA/L-DOPA, mDS, t_52_ = 7.038, ****p < 0.0001; Oil/L-DOPA vs RA/L-DOPA, t_52_ = 3.953, **p < 0.01. (**C**) Representative images show pERK (green), CALB1 (blue), ALDH1A1 (purple), TH (red) co-staining in the coronal sections of 3–month–old *Pitx3*^ak/ak^ mice (n = 5 animals per genotype per group), which were pretreated with corn oil or RA for seven days and then subjected to L-DOPA (10 mg/kg bodyweight) or saline injection. Solid arrowheads point to the dDS. Scale bar: 1000 µm. (**D**) Western blots show pERK, pT34 and pT75 DARPP32 levels in 3–month–old oil or RA–pretreated *Pitx3*^ak/ak^ mice after L-DOPA or saline injection. GAPDH was used as the loading control. (**E–G**) Scatter plots show pERK (**E**), pT34 (**F**) and pT75 (**G**) DARPP32 ratios in the dorsal striatum of 3–month–old oil or RA–pretreated *Pitx3*^ak/ak^ mice after L-DOPA or saline injection (n = 5 animals per genotype per group). Data were presented as mean ± SEM. Two-way ANOVA followed by Sidak’s multiple comparisons test: pERK, Oil/saline vs RA/saline, t_28_ = 0.3437, ns; Oil/saline vs Oil/L-DOPA, t_28_ = 14.04, ****p < 0.0001; RA/saline vs RA/L-DOPA, t_28_ = 10.63, ****p < 0.0001; Oil/L-DOPA vs RA/L-DOPA, t_28_ = 3.056, *p < 0.05. For pT34, Oil/saline vs RA/saline, t_28_ = 0.6084, ns; Oil/saline vs Oil/L-DOPA, t_28_ = 6.767, ****p < 0.0001; RA/saline vs RA/L-DOPA, t_28_ = 2.749, ns; Oil/L-DOPA vs RA/L-DOPA, t_28_ = 3.409, *p < 0.05.

Although *Pitx3*^ak/ak^ mice fail to develop most nDA neurons^25,26^, they exhibit normal general motor activity^32^. On the other hand, administration of L-DOPA induces repetitive dyskinetic climbing movements in *Pitx3*^ak/ak^ mice, which stand vertically, place two to four paws on the wall, and move limbs up and down repetitively^27,28^. Consistent with prior reports, we observed the same stereotypic, repetitive climbing movements in L-DOPA–treated *Pitx3*^ak/ak^ mice in a L-DOPA dose–dependent manner (Fig. 8A). To examine whether a lack of RA and MOR1 expression in the dorsal striatum contributes to these stereotypic, L-DOPA–induced behaviors, we pretreated *Pitx3*^ak/ak^ mice with dietary RA for seven days before L-DOPA injection. We found that pretreatment of RA effectively reduced the duration and occurrence of L-DOPA-induced repetitive climbing movements in *Pitx3*^ak/ak^ mice (Fig. 8B, Additional File 2: Video). These results demonstrate a beneficial effect of RA supplementation in suppressing L-DOPA–induced abnormal, repetitive activities which may model iatrogenic dyskinesias or compulsions.

### RA supplementation improves MOR1 signaling in the dorsal striatum of *Pitx3*^ak/ak^ mice

A substantial increase of phosphorylated ERK (pERK) is observed in the dorsal striatum of *Pitx3*^ak/ak^ mice treated with L-DOPA^27^. Accordingly, we found through immunostaining and western blot analyses (Fig. 8C–E) that pretreatment with RA for seven days suppressed the abnormal increase of pERK in the dorsal striatum of L-DOPA–treated *Pitx3*^ak/ak^ mice. Since activation of MOR1 signaling inhibits cAMP production and the downstream protein kinase A (PKA)–dependent pathways^21^, a lack of MOR1 may disinhibit PKA activity. In line with this notion, the ratio of phosphorylated threonine 34 (pT34) of dopamine– and cAMP–regulated phosphoprotein Mr 32 (DARPP32), a key PKA substrate in SPNs^33^, was substantially increased in the dorsal striatum of L-DOPA–treated *Pitx3*^ak/ak^ mice, while RA treatment reduced the pT34 ratio (Fig. 8C,F). On the other hand, the ratio of PKA–independent phosphorylation of T75 (pT75) of DARPP32 did not change in response to L-DOPA or RA treatment (Fig. 8D,G). These results suggest that RA supplementation not only increases MOR1 expression but effectively improves MOR1 signaling in the dorsal striatum of *Pitx3*^ak/ak^ mice to maintain control over PKA and ERK pathways. Through this mechanism, dietary RA may contribute to the alleviation of L-DOPA–induced compulsive movements in these animals.

## Discussion

In this study, we reveal a previously unknown function of ALDH1A1 in maintaining postsynaptic MOR1 expression and activity in the dorsal striosomal neurons of postnatal and adult animals through transsynaptic RA signaling. We provide evidence that dietary supplementation of RA can restore MOR1 levels in the *Aldh1a1*^−/−^ mice, as well as upregulate MOR1 signaling and mitigate L-DOPA–induced dyskinetic movements in *Pitx3*^ak/ak^ mice. Our findings support a beneficial role of dietary RA supplementation in alleviating LID.

RA is required for striosomal neuron genesis during embryonic development^30^. The endogenous RA produced by ALDH1A3 in the striosomal neuron precursors activates RARβ signaling, and leads to the expansion of progenitor cells, and eventually to the formation of striosome compartments, whereas RARβ–deficiency causes selective loss of striosome compartmentation and MOR1 expression in the dorsal striatum^30^. Although ALDH1A1 is also expressed by nDA neurons in the same developmental stages, no apparent alterations of MOR1 expression and striatal compartmentation was found in the P0 *Aldh1a1*^−/−^ pups, suggesting that ALDH1A1 does not play a major role in the genesis of MOR1–positive striosomal neurons during embryonic development. Instead, ALDH1A1 is crucial in maintaining MOR1 levels in the postsynaptic SPNs in the dorsal striatum of postnatal and adult animals. By contrast, the expression of MOR1 in the ventral striatum was less affected by the loss of ALDH1A1, suggesting the presence of alternative mechanisms in regulating MOR1 expression outside of the dorsal striatum. Since ALDH1A1 resides in both soma and axon terminals, it may produce and/or release RA within efferent striatal regions, to achieve high local RA concentrations among targeted postsynaptic SPNs. In line with this notion, higher levels of MOR1 staining were found in the dorsal striatal regions that are heavily innervated by the ALDH1A1–positive fibers. A lack of ALDH1A1 also changes the GABA and dopamine transmission in the striatum^9,34^. However, since the supplement of RA fully rescued the expression of MOR1 in *Aldh1a1*^−/−^ mice, our findings argue that RA produced by ALDH1A1 plays the main role in promoting MOR1 expression in the dorsal striatum.

RA may enhance MOR1 protein expression through activating RARβ–mediated transcription, since *Mor1* mRNA was also upregulated after RA treatment. Nonetheless, we cannot exclude the possibility that RA directly regulates the synthesis of postsynaptic proteins in the dendrites^35^, resulting in increased postsynaptic MOR1 protein levels. It is worthwhile to point out that RA only partially rescued MOR1 expression in the dorsal striatum of adult *Pitx3*^ak/ak^ mice, suggesting that the presence of nDA neuron innervation may also be needed for maintaining normal expression of MOR1. As RA supplementation also partially rescues the embryonic developmental defects of nDA neurons in *Pitx3*^ak/ak^ embryos^24^, it will be interesting to determine whether early treatment with RA starting at embryonic stages could further improve MOR1 expression in the adult animal.

RA treatment effectively alleviated L-DOPA–induced dyskinetic movements in *Pitx3*^ak/ak^ mice. Excessive ERK activation is suggested to play a causal role in the development of LID, as inhibition of ERK activity reduces dyskinesia^36^. A lack of MOR1 signaling, which inhibits dopamine receptor 1 (DRD1)–mediated activation of adenylyl cyclase and thereby reduces the production of cAMP in the SPNs^37^, could be expected to disinhibit PKA activity. This disinhibition may result in aberrant phosphorylation of DARPP32 and ERK to generate LID^38,39^. Therefore, RA supplementation may reduce LID-like phenotypes in *Pitx3*^ak/ak^ mice through up-regulation of MOR1 signaling and down-regulation of excessive PKA and ERK activation in the SPNs. Indeed, RA treatment also reduced the L-DOPA–induced abnormal phosphorylation of DARPP32 by PKA as well as ERK activity in the striatum of *Pitx3*^ak/ak^ mice.

The decrease of MOR1 expression can be an effector resulted from the PD-related reduction of ALDH1A1 expression and the degeneration of ALDH1A1-positive dopaminergic neurons. The decrease of MOR1 can also alter the synaptic transmission of striatal neurons through the inhibitory G protein-coupled intracellular signaling transduction pathways and contribute to the clinical symptoms. It is worth pointing out that systematic administration of MOR1 antagonists could hit multiple targets in the brain and thereby make it difficult to interpret the behavioral data. Future studies will need to develop new strategies to specifically inhibit MOR1 expression in the dorsal striatum of PD models. Clinically, the ligand binding of MOR1 or KOR1 is downregulated in the striatum of PD patients under chronic L-DOPA administration^40,41^. MOR1 levels are also altered in striatum and globus pallidus interna (GPi) of monkey LID models^42,43^. And yet, reduced MOR1 activity does not clearly lead to LID as MOR1 antagonists cyprodine and ADL5510 reduce LID but preserve the anti-PD efficacy of L-DOPA in the monkey models^44,45^. However, another opioid receptor antagonist naloxone shows no such effect in PD patients^46,47^. These conflicting results may reflect differential alterations of MOR1 signaling in different brain regions between patients and the monkey models^48^. It is also unclear whether alterations of MOR1 expression in PD and LID are causal to the disease or serve as compensatory responses^44,49^. New research continues to clarify the complex balance in protein expression which defines healthy striatal compartmentation^50^. Therefore, a regionally selective application of MOR1 agonists or antagonists could more effectively alleviate LID. In this regard, systemic administration of RA via diet appears to selectively restore a natural pattern of MOR1 expression in the dorsal striatum, and it might therefore provide a fortuitous avenue to achieve a meaningful, regional effect and mitigate the side effects of L-DOPA therapy.

In conclusion, we demonstrated for the first time that the presence of ALDH1A1 in nDA neurons is required for maintaining normal postsynaptic MOR1 levels in the dorsal striosomes of postnatal and adult animals. We further found that dietary supplementation of RA, a product of ALDH1A1, restores MOR1 expression, implicating nigrostriatal, transsynaptic RA signaling in regulating postsynaptic MOR1 levels in dorsal striatum. More importantly, we showed that RA supplementation had beneficial effects in mitigating L-DOPA–induced dyskinetic movements through promoting MOR1 activity in an nDA neuron-depleted mouse model, suggesting potential therapeutic applications for dietary RA or related compounds in treating LID.

## Methods

### Animal subjects

*Aldh1a1*^−/−^ (Stock # 012247)^51^ and *Pitx3*^ak/ak^ (Stock # 000942)^25,26^ mice were obtained from the Jackson Laboratory (Bar Harbor, ME), and backcrossed with C57BL/6J mice for more than five generations. Nr4a1–EGFP139Gsat (Nr4a1–GFP) (Stock # 036737-UCD)^29^ transgenic mice were obtained from MMRRC and backcrossed with C57BL6J mice for more than five generations. Nr4a1–GFP mice were crossbred with *Aldh1a1*^−/−^ and *Pitx3*^ak/ak^ mice to generate Nr4a1–GFP/*Aldh1a1*^−/−^ and Nr4a1–GFP/*Pitx3*^ak/ak^ bigenic mice. All mice were housed in a 12-hour-light/dark cycle. All mice were fed either regular diet or corn oil/retinoic acid–supplemented diet ad libitum. All mouse work is approved by the Institutional Animal Care and Use Committees of the National Institute on Aging, NIH.

### Genotyping

Genomic DNA was prepared from tail biopsy using DirectPCR Lysis Reagent (Viagen Biotech Inc.) and subjected to PCR amplification using specific sets of PCR primers for each genotype. *Aldh1a1*^−/−^ mice were genotyped with primers of ALDH1A1muF (5′-CTA TCG CCT TCT TGA CGA GTT CTT-3′), ALDH1A1muR (5′-CCT TGT ACA TCT TAA CGG TGC ACA-3′), ALDH1A1wtF (5′-TAA AGA CCT GGA TAA GGC CAT CA-3′), and ALDH1A1wtR (5′-ACG GTG CAC AAA ATA AAC ATC TG-3′). Nr4a1–GFP mice were genotyped at birth by fluorescence of the thymus, tails and ears using a hand–held LED light source. *Pitx3*^*ak/ak*^ were genotyped based on the appearance of small, closed eyes due to abnormal lens development^25^. *Pitx3*^+/+^ and *Pitx3*^*ak/+*^ mice showed normal eye phenotype, and thereby were grouped together as controls.

### Immunohistochemistry and light microscopy

Mice were anesthetized with ketamine and then transcardially perfused with a 4% formaldehyde/PBS solution as described previously^52^. The brains were extracted and post fixed in 4% formaldehyde overnight, then submerged in 30% sucrose for 24 hours at 4 °C for later sectioning. Series of 40 μm sections were collected using a cryostat (Leica Biosystems, Richmond IL). The sections were blocked in a 10% normal donkey serum, 1% bovine serum albumin, 0.3% Triton X-100, PBS solution for 1 hour at room temperature on an orbital shaker at low speed. The sections were then incubated in a primary antibody/PBS solution over two nights as follows: rabbit anti-MOR1 (1:3,000; Immunostar), goat anti-ALDH1A1 (1:75, R&D system), or rabbit anti-ALDH1A1 (1:1000, Sigma-Aldrich), or mouse anti-TH (1:1000, Immunostar), or chicken anti-TH (1:1000, Immunostar) or chicken anti-GFP (1:1000, ab5450, Abcam), or mouse anti-Calbindin (1:200, Millipore). Sections were washed three times in PBS before being incubated in secondary antibody solutions with Alexa Fluor 405- or Alexa 488- or Alexa Fluor 546–, or Alexa Fluor 633-conjugated secondary antibody (1:500, Invitrogen) at room temperature for 1 hour. Following three washes in PBS, sections were mounted onto subbed slides, and coverslipped with mounting media (ProLong® Gold Antifade Mountant, Life technology). Stained sections were imaged using a laser scanning confocal microscope (LSM 780; Zeiss). The paired images in the figures were collected at the same gain and offset settings. After collection, processing was applied uniformly to all paired images. The images were either presented as a single optic layer from individual fields or displayed as maximum intensity projections to represent confocal stacks.

### Stereotaxic viral injection

Stereotaxic virus injections were conducted on 3 to 6–month–old male or female mice. The animal was initially anesthetized in an induction chamber. It was then head-fixed on the stereotaxic stage and maintained under anesthesia by a nose-cone connected to oxygen and isoflurane vaporizer (Vet Equip, Inc) for anesthesia, and a scavenger system for collection of exhaled CO_2_ and isoflurane to a charcoal filter. The O_2_ was kept at 1–2 liters per min and the isoflurane at 2–3% during surgery. A total volume of 500 nl of virus solution was injected unilaterally or bilaterally into SNpc (coordinates used, AP: −1.5 mm, ML: ±0.9 mm from Lambda DV: −4.1 mm from exposed dura mater) or dorsal striatum (coordinates used, AP: +0.3 mm, ML: ±2.5 mm from Bregma, DV: −3.1 mm from exposed dura mater). Virus solution was injected at an infusion rate of 100 nl/min and withdrawn 10 min after the end of injection. Following virus injection, the scalp was sutured, and mice were returned to their home cages. Mice were used for experiments at least 4 weeks after virus infusion.

### Image analysis

For the quantitative assessment of various marker proteins in striatum, the dorsal striatum (DS) striosomes were chosen at random from three or four mice per group and imaged by a 10 × objective. A single optical layer was taken using identical settings and exported to ImageJ (NIH) for imaging analyses. Images were converted to an 8-bit color scale (fluorescence intensity from 0 to 255) using ImageJ. Areas of interest, striosomes and adjacent matrix, were first selected by Polygon or Freehand selection tools and then subjected to measurement by mean optical intensities. The mean intensity for the background area was subtracted from the selected area to determine the net mean intensity. Three sequential coronal sections (between Bregma 0.86–1.18 mm) were analyzed per animal.

### Western blot

Tissue or neurons were lysed in 50 mM Tris-HCl pH7.6, 150 mM NaCl, 2 mM EDTA, 2% SDS or RIPA buffer (Sigma Aldrich) supplemented with complete protease inhibitor mixture (Roche Applied Biosciences) and phosphatase inhibitor mixture (Pierce Biotechnology). Proteins were separated by 4–12% NuPage Bis-Tris PAGE (Invitrogen) and transferred to nitrocellulose membranes. Antibodies specific for MOR1 (1:1000, Millipore), DARPP32-pT75 (1:1000, Cell Signaling), DARPP32-pT34 (1:1000, Cell Signaling), DARPP-32 (1:1000, Cell Signaling), ALDH1A1 (1:1000, Sigma-Aldrich), pERK (1:1000, Cell Signaling), ERK (1:1000, Cell Signaling) were used. Anti-GAPDH antibody (1:1000, Sigma–Aldrich), Anti-β-actin antibody (1:1000, Sigma–Aldrich) were used to adjust equal amounts of protein loading into each well. The secondary antibodies conjugated with Alexa Fluor® 680 or Alexa Fluor® 800 were purchased from Abcam. Protein bands were detected using Odyssey CLx Imaging System (LI-COR) and quantified using the Scion Image System.

### Endogenous retinoic acid measurement by ELISA

The dorsal and ventral striatal tissues from 12-month-old control and *Aldh1a1*^−/−^ mice were dissected under yellow light. Tissues were snap frozen in dry ice immediately after harvest and stored at −80 °C until assay. Within one week of harvest, tissues were weighed and homogenized in 100 volumes of ice-cold 0.9% saline with Dounce homogenizer (5 strokes). The homogenates were centrifuged at 12,000 × *g* for 5 min at 4 °C, and the supernatants were collected. The supernatants were diluted 10 times and assessed for the endogenous retinoic acid concentration by using mouse retinoic acid ELISA kit (MyBioSource) according to the manufacturer’s instructions. The level of retinoic acid in each sample was assayed in quadruplicate, and the averaged value was normalized against the concentration of total protein present in the sample, which was determined by bicinchoninic acid (BCA) assay kit (Thermo Fisher Scientific).

### Mouse behavior tests

#### Open-field test

Mice removed from the light cycle were placed in the dark room for 30 min before being placed in the chamber for Open-field test using Flex-Field activity system (San Diego Instruments, CA) as described previously^52^.

#### L-DOPA–induced repetitive movement test

Following the procedures described in the previous studies^27,28^, we placed individual mice in a transparent Perspex box (L20 cm/W20 cm/H30 cm) and video-recorded their movements before and after injection of L-DOPA or saline. A 10-min video taken 30 min after L-DOPA or saline injection was analyzed off–line. The videos were replayed at slow motion or frame by frame when necessary. Behavior was scored using previously published criteria^27^; in brief, the duration of individual dyskinetic paw and trunk movements was counted manually and added together to get the total dyskinetic duration. All behavioral scoring was done by reviewers who were blind to treatment.

### Laser Capture Microdissection (LCM) and quantitative reverse transcription polymerase reaction (qRT-PCR)

Mouse brains of different genotypes and treatments were quickly dissected out and frozen down at −80 °C. The frozen mouse brains were sectioned with a cryostat at −20 °C and loaded onto the PAN membrane frame slide (Applied Biosystems, Foster City, CA). The dorsal striatum was dissected by the LCM with the ArturusXT micro-dissection system (Applied Biosystems) based on the anatomic landmarks in the striatum such as corpus callosum (CC), lateral ventricle (LV) and nucleus accumbens (Acb). The total RNAs were extracted with the PicoPure Isolation kit (Applied Biosystems) and the cDNAs were synthesized with the First Strand kit (QIAGEN, Valencia, CA) from equal amounts of total RNAs in the comparison groups. The SYBR Green real-time PCR detection method was used to quantify the Aldh1a1 and opioid receptors as well as the peptides. All the primers used in the qPCR were from QIAGEN and tested by the manufacturer. Gene expression was calculated as fold change normalized to β-actin.

### Drugs

l-3,4-dihydroxyphenylalanine methyl ester hydrochloride (L-DOPA) and benserazide hydrochloride (peripheral L-DOPA decarboxylase inhibitor) were purchased from Sigma-Aldrich (St. Louis, MO). These drugs were dissolved in 0.9% saline and delivered to the mice via intraperitoneal (IP) injection. L-DOPA was always injected together with 5 mg/kg benserazide to inhibit peripheral DOPA decarboxylase. To avoid potential dopamine sensitization effects, each of these drugs was injected into a separate group of naïve mice. For dose-response experiments, each dose was also tested in a separate group of naïve mice. The injection was made between 9 AM and 4 PM. As described previously^24^, all-trans-retinoic acid (RA) was dissolved in corn oil and mixed with regular mouse food pellets to a final concentration of 0.25 mg/g food. RA–supplemented diet was prepared fresh daily. The regular diet mixed with corn oil alone served as the control.

### Statistics

GraphPad Prism 6 (GraphPad Software, La Jolla, CA) was used for all statistical tests. Unpaired t-tests and ANOVA (one-way or two-way) tests were used, with significant ANOVA followed by post hoc tests. Data was presented as mean ± SEM. *P < 0.05; **p < 0.01; ***p < 0.001, ****p < 0.0001.

### Ethics approval and Consent to participate

All animal procedures conformed to the NIH guide for the ethical care and use of laboratory animals. Animal protocols were approved by the Institutional Animal Care and Use Committee of National Institute on Aging.

## Supplementary information

Supplementary materials

Supplementary video clip

## Data Availability

All supporting data are available upon request.
