# Supplementary material for: ALDH1A1 regulates postsynaptic μ–opioid receptor expression in dorsal striatal projection neurons and mitigates dyskinesia through transsynaptic retinoic acid signaling
Source: Sci Rep. 2019 Mar 5;9:3602. doi: 10.1038/s41598-019-40326-x (PMC6401150; doi:10.1038/s41598-019-40326-x)
Supplement: Supplementary file 1 — Supplementary materials [file 41598_2019_40326_MOESM1_ESM.pdf]

ALDH1A1 regulates postsynaptic μ–opioid receptor expression in the dorsal striatal projection neurons and mitigates dyskinesia through transsynaptic retinoic acid signaling

Jing Pan[1, 2], Jia Yu[3], Sarah Hawes[2], Junbing Wu[2], Lixin Sun[2], Chengsong Xie[2], Lisa Chang[2], Sara Saez–Atienzar[2], Wang Zheng[2, #], Justin Kung[2], Jinhui Ding[4], Weidong Le[5], Shengdi Chen[1, *], and Huaibin Cai[2, *, ^]

Supplementary figures, figure legends, and video legend

Fig. S1

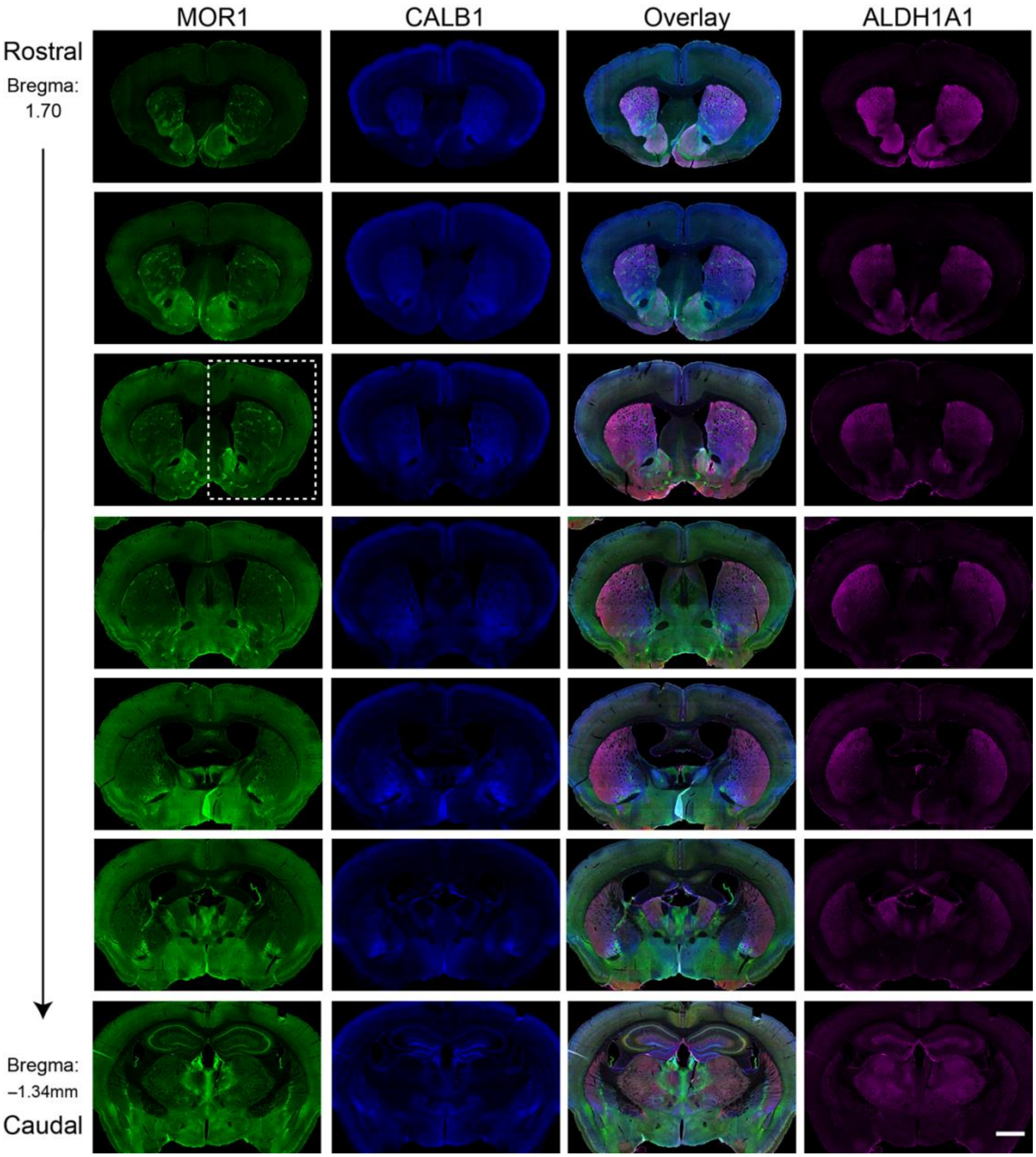

Fig. S1 Uneven distribution of MOR1 and ALDH1A1staining in the dorsal striatum. Images show MOR1, CALB1, and ALDH1A1 co-staining in sequential coronal sections evenly sampled across the entire striatum of 3–month–old *Aldh1a1*$^{+/+}$ mice. MOR1–positive striosomes are mainly distributed in the rostral-dorsal portions of dorsal striatum, which are under more intense innervation of ALDH1A1–positive axons. The boxed area was highlighted in Fig. 1b. Scale bar: 1000μm.

Fig. S2

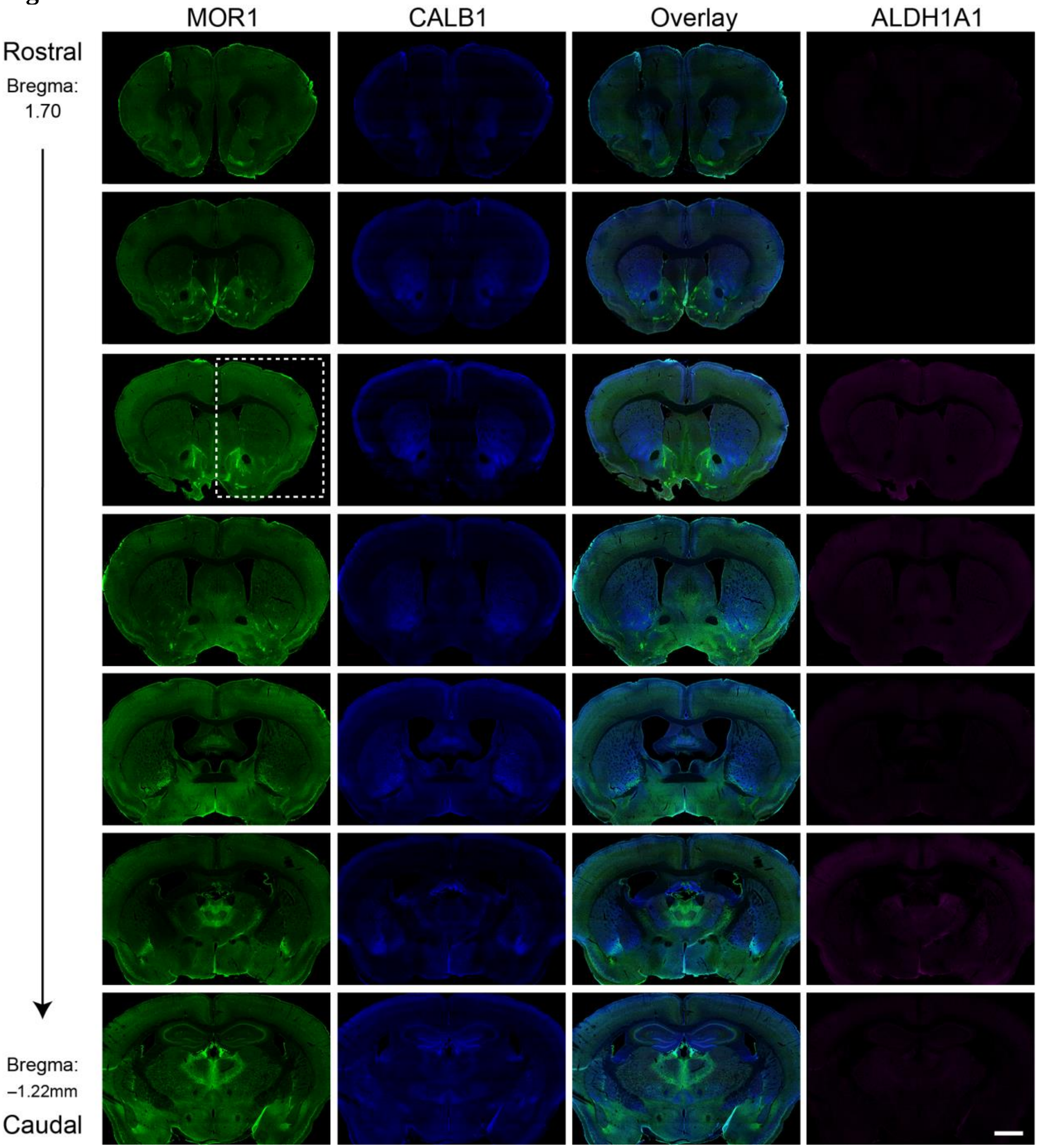

Fig. S2 Substantial reduction of MOR1 staining in the dorsal striatum of *Aldh1a1*$^{-/-}$ mice. Images show MOR1, CALB1, and ALDH1A1 co-staining in sequential coronal sections evenly sampled across the entire striatum of 3–month–old *Aldh1a1*$^{-/-}$ mice. MOR1 staining was severely reduced in the dorsal portions of dorsal striatum. The boxed area was highlighted in Fig. 1b. Scale bar: 1000μm.

Fig. S3

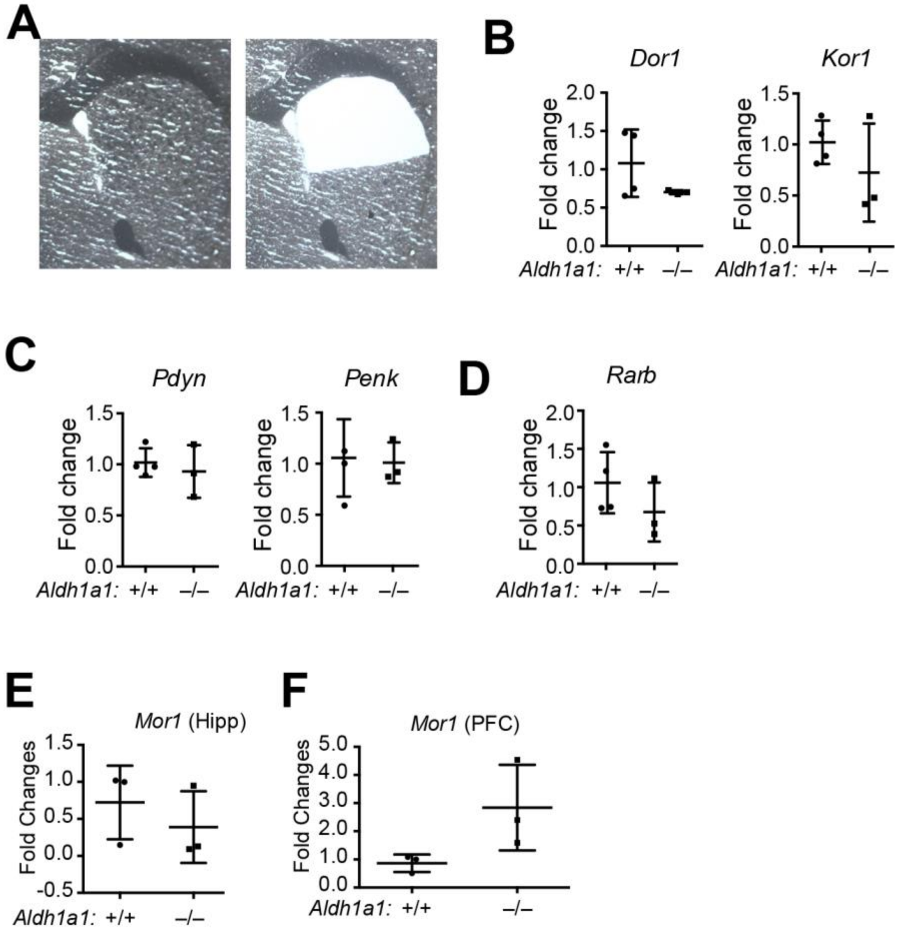

Fig. S3 Quantification of endogenous opioid receptor and ligand mRNA expression in the dorsal portion of dorsal striatum and other brain regions. (A) Images show coronal striatal sections before and after removal of DS by laser microdissection for the follow-up qRT–PCR analyses. (B–D) Scatter plots depict the expression of endogenous opioid receptor Dor1 and Kor1 (B), ligand *Pdyn* and *Penk* (C), and retinoic acid receptor *RAR*β (D) mRNAs in the DS of 3–month–old *Aldh1a1*$^{+/+}$ (n=4) and *Aldh1a1*$^{-/-}$ (n=3) mice by qRT-PCR. Data were presented as mean ± SEM. Unpaired t test was used for statistical analysis, followed by *post hoc* tests. No significant difference was found. (E, F) Expression of *Mor1* mRNA in the hippocampus (Hipp) and prefrontal cortex (PFC) of 3–month–old *Aldh1a1*$^{+/+}$ (n=3) and *Aldh1a1*$^{-/-}$ (n=3) mice by qRT-PCR. Data were presented as mean ± SEM. Unpaired t test was used for statistical analysis, followed by *post hoc* tests. No significant difference was found.

Fig. S4

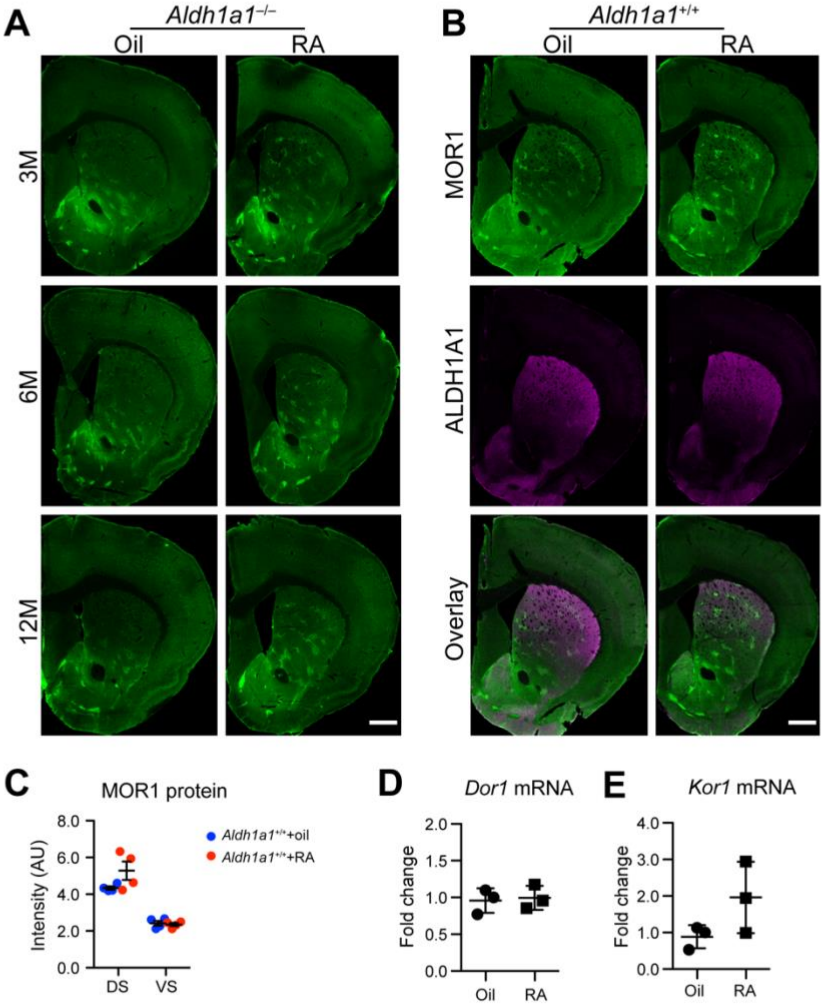

Fig. S4 RA treatment increases MOR1 expression in the dorsal striatum of *Aldh1a1*$^{-/-}$ mice at different age. (A) MOR1 staining in the selective striatal coronal sections of *Aldh1a1*$^{-/-}$ mice treated with oil or RA at different age. Scale bar: 1000μm. (B) MOR1 and ALDH1A1 co-staining in the selective striatal coronal sections of 3-month-old *Aldh1a1*$^{+/+}$ mice treated with oil or RA. Scale bar: 1000μm. (C) Scatter plot shows the signal intensities of MOR1 staining in the DS and VS of *Aldh1a1*$^{+/+}$ treated with oil or RA (n=4 animals per genotype per age group, 3 sequential coronal sections per animal). Data were presented as mean ± SEM. Two-way ANOVA followed by Sidak's multiple comparisons test, no significant difference. (D, E) Expression of *Dor1* (D) and *Kor1* (E) mRNA in the DS of 3–month–old *Aldh1a1*$^{-/-}$ (n=3) mice treated with oil or RA by qRT-PCR. Data were presented as mean ± SEM. One-way ANOVA was used for statistical analysis, followed by *post hoc* tests. No significant difference was found.

Fig. S5

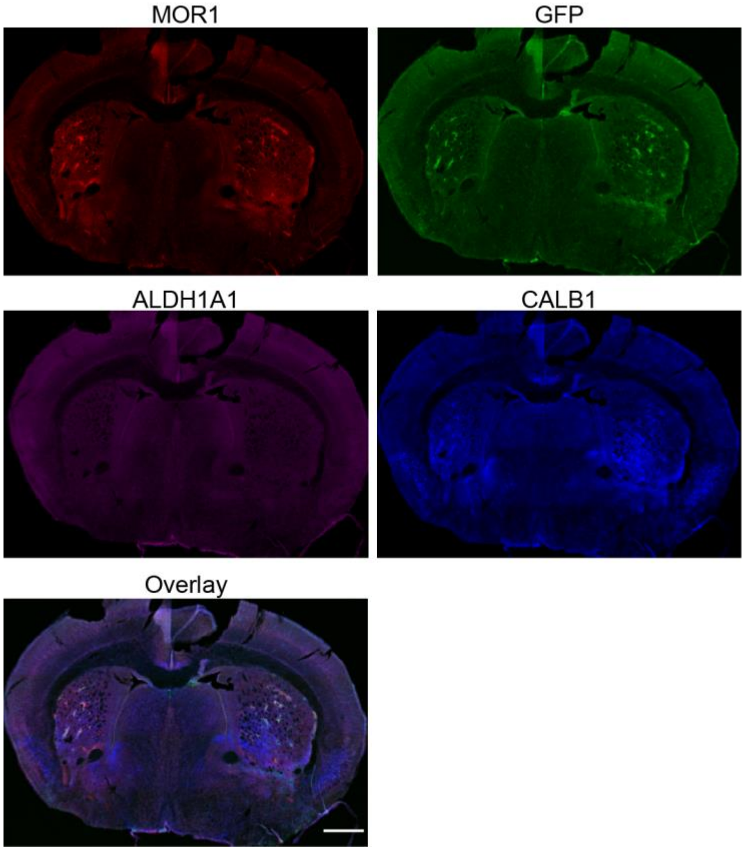

Fig. S5 No apparent alterations of MOR1 expression pattern and striosome/matrix compartmentation in P0 *Nr4a1-GFP/Pitx3*$^{ak/ak}$ mice. Representative images show MOR1, GFP, ALDH1A1, and CALB1 co-staining in the striatal coronal sections P0 Nr4a1-GFP transgenic pups with *Pitx3*$^{ak/ak}$ background. Scale bar: 1000μm.

Fig. S6

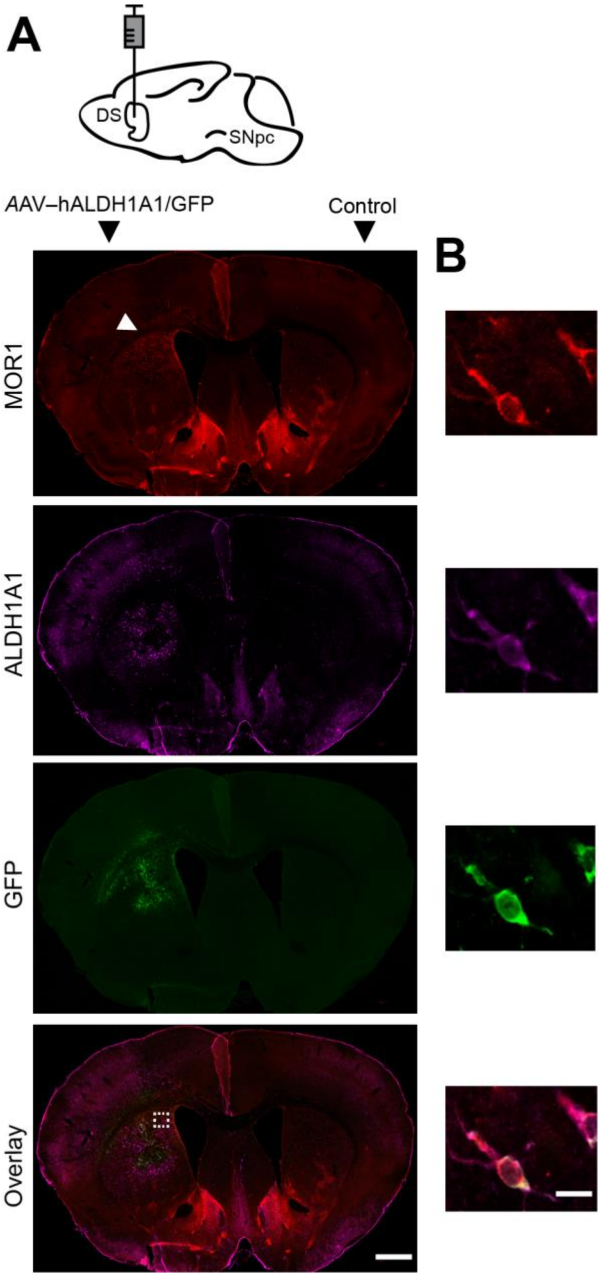

Fig. S6 Ectopic expression of ALDH1A1 in the dorsal striatum increases MOR1 levels in *Pitx3*$^{\mathbf{ak/ak}}$ SPNs. (A) Cartoon indicates the needle placement in one side of dorsal striatum for stereotaxic injection of AAVs co-expressing human ALDH1A1 and GFP[1]. Representative images show MOR1, ALDH1A1, and GFP co–staining in the striatal coronal sections of 3-month-old *Pitx3*$^{ak/ak}$ mice injected with AAVs at one hemisphere. Arrowhead points to the MOR1 staining in the dDS in the hemisphere injected with *hALDH1A1*–expressing AAVs. Scale bar: 1000µm. (B) Enlarged images from the boxed region in (A) show co-expression of MOR1, ALDH1A1, and GFP in striatal neurons. Scale bar: 50µm.

Fig. S7

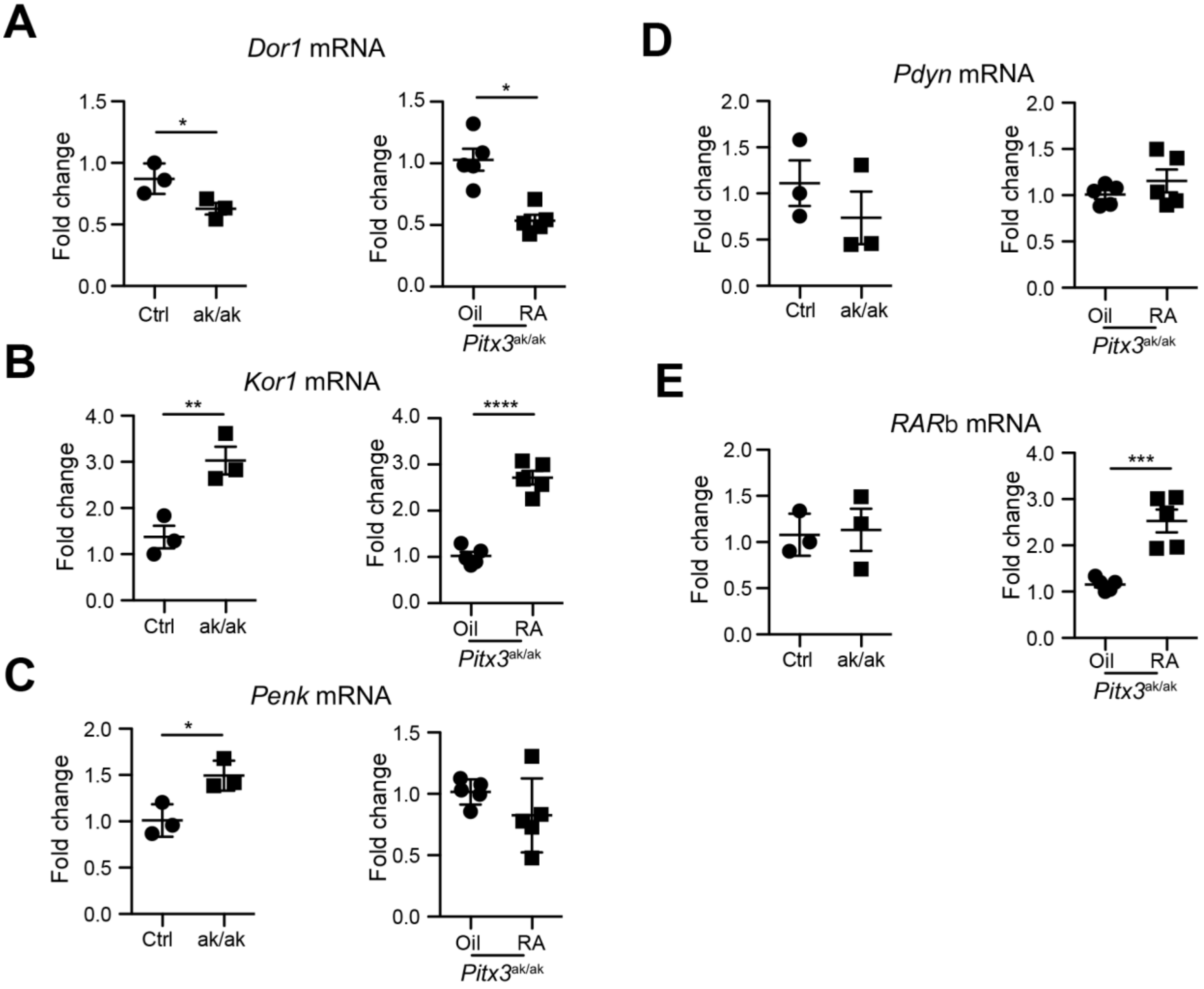

Fig. S7 Alterations of endogenous opioid receptor and peptide mRNA as well as retinoic acid receptor mRNA expression in the dDS of *Pitx3*$^{ak/ak}$ mice. Scatter plots compare *Dor1* (A), *Kor1* (B), *Penk* (C), *Pdyn* (D), *RARβ* (E) mRNA levels in the DS of 3–month–old control (Ctrl) and *Pitx3*$^{ak/ak}$ mice (left panel), as well as 3–month–old *Pitx3*$^{ak/ak}$ mice after treated with oil or RA for seven days (right panel). Data were presented as mean ± SEM. Unpaired t test, (A) t_4=2.838, p=0.023 (left panel); t_8=4.928, p=0.0006 (right panel). (B) t_4=4.296, p=0.0063 (left panel); t_8=9.901, p<0.0001 (right panel). (C) t_4=3.515, p=0.00123 (left panel); t_8=1.350, p=0.1070 (right panel). (D) t_4=0.9960, p=0.1871 (left panel); t_8=1.109, p=0.1499 (right panel). (E) t_4=0.2005, p=0.4254 (left panel); t_8=5.462, p=0.0003 (right panel).

Supplementary Video

Video clip shows a pair of 4–month–old male littermate *Pitx3*$^{ak/ak}$ mice in a Perspex box after treated with RA or oil for 7 days and then with L-DOPA. The dyskinetic movements consist with a vertical position of trunk supported by 1 or 2 hind limbs, and the repeated waving of forelimbs along the cage walls. The recording started 30 min after injecting the mice with 10mg/kg L-DOPA together with 5mg/kg benserazide.
